# Modulations of the executive control network by stimulus onset asynchrony in a Stroop task

**DOI:** 10.1186/1471-2202-14-79

**Published:** 2013-07-31

**Authors:** Emily L Coderre, Walter J B van Heuven

**Affiliations:** 1 School of Psychology, University of Nottingham, University Park, Nottingham NG7 2RD, UK; 2 Department of Neurology, Johns Hopkins University School of Medicine, 1629 Thames St. Suite 350, Baltimore, MD 21231, USA

**Keywords:** fMRI, Stroop task, Stimulus onset asynchrony, Cognitive control

## Abstract

**Background:**

Manipulating task difficulty is a useful way of elucidating the functional recruitment of the brain’s executive control network. In a Stroop task, pre-exposing the irrelevant word using varying stimulus onset asynchronies (‘negative’ SOAs) modulates the amount of behavioural interference and facilitation, suggesting disparate mechanisms of cognitive processing in each SOA. The current study employed a Stroop task with three SOAs (−400, -200, 0 ms), using functional magnetic resonance imaging to investigate for the first time the neural effects of SOA manipulation. Of specific interest were 1) how SOA affects the neural representation of interference and facilitation; 2) response priming effects in negative SOAs; and 3) attentional effects of blocked SOA presentation.

**Results:**

The results revealed three regions of the executive control network that were sensitive to SOA during Stroop interference; the 0 ms SOA elicited the greatest activation of these areas but experienced relatively smaller behavioural interference, suggesting that the enhanced recruitment led to more efficient conflict processing. Response priming effects were localized to the right inferior frontal gyrus, which is consistent with the idea that this region performed response inhibition in incongruent conditions to overcome the incorrectly-primed response, as well as more general action updating and response preparation. Finally, the right superior parietal lobe was sensitive to blocked SOA presentation and was most active for the 0 ms SOA, suggesting that this region is involved in attentional control.

**Conclusions:**

SOA exerted both trial-specific and block-wide effects on executive processing, providing a unique paradigm for functional investigations of the cognitive control network.

## Background

The term ‘cognitive control’ refers to a broad array of cognitive situations in which distracting information must be ignored, a habitual response must be overcome, or one must switch between varying mental sets. In the cognitive psychology literature, cognitive control is labeled more formally as ‘executive function’, a category which spans a number of cognitive functions such as working memory, response selection and/or suppression, and conflict detection and resolution. A vast amount of literature has been dedicated to understanding the cognitive and neural mechanisms of these various aspects of executive control, and with the emergence of neuroimaging technologies such as positron emission tomography (PET), magnetoencephalography (MEG) and functional magnetic resonance imaging (fMRI), this literature has grown enormously.

Previous work using fMRI has identified an extensive network of executive control consisting of regions across the prefrontal and parietal cortices that participate in a range of cognitive functions. For example, the rostral cingulate zone (RCZ; located along the borders of Brodmann areas (BAs) 6, 8, 32 and 24 in the medial frontal cortex) is thought to be involved in performance monitoring [1]. A subset of this region, the anterior cingulate cortex (ACC: Brodmann areas (BAs) 24/32) is clearly involved in executive control but is highly debated regarding its precise function, being implicated in processes such as conflict monitoring [2-4], top-down regulation of conflict [5], and anticipatory adjustments in control [6], to name a few (see [1,7,8] for reviews). Other frontal areas such as the middle and inferior frontal gyri, including the dorsolateral prefrontal cortex (DLPFC; BAs 9/46) are involved in conflict processing and regulation of executive control [2-4,7]. The left inferior frontal gyrus (LIFG) is believed to implement cognitive control via suppression of irrelevant semantic information [9-12], while the right inferior frontal gyrus (RIFG) is involved in inhibitory control, specifically response inhibition [13-16]. The inferior (BAs 39/40) and superior (BA 7) parietal lobes are involved in top-down visuospatial control of attention towards the task-relevant target or attribute [17-20]. Other areas of the prefrontal cortex such as the premotor (BA 6) and frontopolar (BA 10) cortices are also involved, as are subcortical structures such as the thalamus and caudate (e.g. [1,21,22]; see [23,24] for meta-analyses).

Although a number of alternative theories exist regarding the precise function of these structures, especially the ACC (e.g. [6,7,25]), DLPFC (e.g. [26]), and RIFG (e.g. [15,27]), these areas are reliably activated for a spectrum of executive control functions, including working memory, cognitive flexibility, vigilance or sustained attention, and – importantly for this study – inhibition of prepotent behaviours and the management of cognitive conflict [23]. The activation and recruitment of this executive control system is also affected by various task parameters, such as the context, magnitude, and nature of cognitive conflict (e.g. [19,28-30]). This malleability of the executive control network highlights its dynamic, moment-to-moment recruitment of different conflict processing strategies. The current study specifically explored how this network is modulated by SOA manipulation in the Stroop task.

This paradigm presents a colour word printed in coloured ink, and asks subjects to ignore the word and respond to its ink colour [31]. Interference arises in incongruent conditions (e.g. *blue* printed in red ink, correct response “red”) due to the conflicting semantic and response information, and longer reaction times (RTs) arise because the automatic process of word reading must be overcome in order to name the colour. The Stroop task recruits the canonical executive control network, generating stronger activation for incongruent trials (e.g. [24,32-35]). Many variations of the Stroop task have been employed with fMRI to investigate the precise function of executive control structures (e.g. [19,36,37]).

One notable variation is stimulus onset asynchrony (SOA) manipulation, which spatially separates the colour and word stimuli (e.g. a coloured rectangle surrounding the word) and presents them at different times in order to gain temporal information on colour and word interference. A ‘negative SOA’ presents the irrelevant stimulus (e.g. the word) before the relevant target stimulus (the colour) at a specific interval. For example, a negative 200 ms SOA (‘-200 ms SOA’) pre-exposes the word for 200 ms before the colour appears. A ‘0 ms SOA’ presents the word and colour simultaneously, as in a traditional Stroop task. Typically, the strongest interference effects (incongruent minus control) occur at −200 ms to 0 ms SOAs [38-41]. Interference is decreased, but remains significant, at negative SOAs out to −400 ms. Facilitation effects (control minus congruent) are generally found for negative SOAs and not for positive SOAs beyond +200 ms [38,39,41,42], but facilitation typically does not differ across negative SOAs [38,41,43,44]. At the 0 ms SOA, some researchers report significant facilitation effects [40] and some do not [38,39,41,42].

The Stroop task is traditionally administered with a verbal response, in which participants name the colour of the ink aloud. A manual response modality, which uses a button-press instead of a vocal response, results in decreased (but still significant) interference effects [45], as well as faster reaction times overall [36,45-47]. SOA has been found to elicit different patterns of interference effects depending on the response modality: vocal responses elicit the maximum amount of interference at a 0 ms SOA (e.g. [38,41]), whereas manual responses shift the peak of interference to the −200 ms SOA due to the faster manual response time [39,40]. In terms of facilitation effects, manual and vocal responses appear to have similar effects on facilitation magnitude across SOAs, with significant facilitation effects for negative SOAs and no facilitation for positive SOAs [38-42].

SOA variation has proven to be a useful manipulation of the Stroop task because it provides temporal information about the speed of processing of the two conflicting stimulus dimensions. To investigate these temporal effects further, recent studies have employed Stroop SOA manipulation with electroencephalography (EEG), to investigate how pre- or post-exposure of the word affects conflict-related ERP components [40,42,48]. These studies have demonstrated that the onset and duration of the N_inc_ or N450 (an ERP component thought to be indicative of conflict detection) is modulated by SOA, and that this component is sensitive to conflict across a variety of task designs and conflict demands. However, the current study is the first to explore the neural effects of Stroop SOA manipulation (using −400 ms, -200 ms, and 0 ms SOAs) on the activation and recruitment of the executive control network using fMRI. Based on prior research, this study addressed three specific cognitive aspects of SOA manipulation.

### SOA effects on neural conflict and facilitation

First, the current study explored how the executive control network in the brain is modulated by conflict and facilitation effects in each SOA. Behaviourally, each SOA generates different magnitudes of interference and facilitation, with maximal interference at simultaneous presentation or short word pre-exposure (i.e. 0 ms or −200 ms) and significant facilitation at negative SOAs [38-40,42]. Furthermore, the N_inc_ ERP component is sensitive to conflict across a variety of task designs and conflict demands [40,42,48]. This modulation of conflict and facilitation effects suggests the participation of different cognitive control mechanisms for each SOA. The primary aim of the current study was therefore to explore how these ‘trial-specific’ effects of SOA affected the activation of the executive control network.

Overall, typical executive control areas of the prefrontal cortex were expected to be elicited by incongruency in the 0 ms SOA (as this was analogous to a traditional Stroop task), such as the RCZ, left middle/medial frontal gyrus (LMFG), and LIFG (e.g. [1,3,12,21-23]), as well as parietal regions such as the left angular gyrus [12,32,49,50] and the inferior/superior parietal lobe [17-20]. Activation in these areas was also expected for the −400 ms and −200 ms SOAs, although with potentially different extents and/or strengths of activation compared to the 0 ms SOA. For example, the executive control network has demonstrated stronger activation in the presence of more conflict (e.g. [29]), so increased behavioural interference in the −200 ms SOA may be reflected in stronger neural recruitment of these areas.

### Response priming effects in negative SOAs

The second topic addressed in the current study regarded the effects of response priming in negative SOAs. Appelbaum et al. [40] have proposed that in negative SOAs, word pre-exposure creates a priming effect by pre-activating response selection. In congruent conditions this accelerates processing time because the subsequently-presented colour matches the pre-activated information, leading to larger behavioural facilitation effects. In contrast, incongruent conditions require more conflict control to overcome or inhibit the primed response, increasing behavioural RTs and interference effects. Increased interference and facilitation effects have been previously documented at the −200 ms SOA, in line with this proposal of response priming effects [39,40]. The current study sought to establish the neural correlates of response priming effects in negative SOAs.

Response priming effects were expected in executive control areas linked to response preparation, such as the DLPFC [51] or supplementary and cingulate motor areas [52]. This activation was predicted to be stronger in the −200 ms SOA, and potentially also the −400 ms SOA, compared to the 0 ms SOA. Furthermore, if the increased behavioural interference in the −200 ms SOA arises from the need to overcome the primed response in incongruent conditions, evidence of response priming may also be observed in areas linked to response inhibition, such as the RIFG [13-16].

### Attentional effects of blocked SOA presentation

Finally, the third aspect of SOA manipulation investigated in this study concerned the effects of blocked SOA presentation. Appelbaum et al. [48] have recently observed different patterns of interference for blocked and mixed SOA presentation. Specifically, temporally-predictable SOAs, as in blocked presentations, may lead to a strategic orientation of attention which could modulate the amount of conflict experienced. In their EEG data, Appelbaum et al. [48] demonstrated that although the N_inc_ tracked the onset of conflict across SOA manipulation, a larger N_inc_ component occurred in the 0 ms SOA when SOAs were blocked, whereas when SOAs were randomized a larger N_inc_ occurred in the −200 ms SOA. In blocks of negative SOAs, the pre-exposed word may have acted as an alerting cue for the upcoming target information, prompting participants to use this cue to strategically orient their attention towards the target stimulus. In contrast, in the 0 ms SOA this strategy could not be used, leading to larger interference effects. Therefore Appelbaum et al. [48] proposed that the temporal predictability of blocked SOAs encourages an attentional orientation strategy. On the other hand, Roelofs [41] has also investigated this issue of blocked versus mixed SOA presentation using a behavioural paradigm and reported that, although overall RTs were affected, no difference in interference patterns occurred between SOA presentation methods. This argues against such a temporal predictability effect, but it may be that the electrophysiological technique used in Appelbaum et al. [48] was more sensitive to strategic attentional effects. The current study therefore also investigated global attentional effects of blocked SOA presentation.

If blocked SOA presentation engages strategic attentional processes, such block-wide SOA effects should be observable in all congruency conditions. The current study investigated these global (i.e. block-wide and conflict-independent) effects of strategic attentional control by first collapsing over congruencies and comparing SOAs, as well as comparing congruency conditions across SOAs (e.g. -400 ms control vs. 0 ms control). Global SOA effects on attentional orientation were expected in areas involved in top-down attentional control such as the right parietal lobe, specifically the angular gyrus (BA 40) and superior parietal lobe (BA 7; [17-20]). Specifically, if subjects use the pre-exposed word in negative SOAs as a temporal cue, activation in these attentional control areas should be enhanced in the −200 ms and −400 ms SOAs compared to the 0 ms SOA.

In summary, the current study employed a Stroop task with negative SOA modulation in fMRI to explore how SOA affects the recruitment and performance of the executive control network. Of specific interest were 1) the effects of SOA on Stroop, interference, and facilitation effects in the brain; 2) response priming effects in negative SOAs; and 3) global effects of blocked SOA presentation on attention.

## Methods

### Participants

Fourteen right-handed participants who had no history of neurological disorder, no colour-blindness, and normal vision, were recruited from the University of Nottingham in the UK. The participants were 10 males and 4 females with a mean age of 25 years (SD = 4.2). This study was approved by the University of Nottingham Medical Ethics Committee. All subjects gave informed written consent according to the ethics guidelines of the University of Nottingham Medical Ethics Committee and in accordance with the Helsinki Declaration. Participants were offered an inconvenience allowance for their participation.

### Design and materials

Three SOAs were used: -400 ms, -200 ms, and 0 ms. Word stimuli consisted of the words ‘red’ , ‘green’ , and ‘blue’ in lowercase letters printed in white ink on a black background. A non-colour, non-word stimulus that matched the visual input of the words (‘%%%%’) was included as a control stimulus, also printed in white ink on a black background. Colour stimuli for both tasks were red, green and blue rectangles surrounding the word stimuli. Participants were asked to respond to the colour of the rectangle by pressing a button on an MRI-compatible button box (right index finger for red, right middle finger for green, right ring finger for blue).

### Procedure

The scanning session lasted approximately 1 hour including set-up, structural image acquisition, and experimental testing. Stimuli were presented using E-Prime. In addition to the three SOAs of the Stroop task, participants also performed a run of a flanker task (data not reported here).^a^ Task order (Stroop or flanker) was counterbalanced between participants. Each run was approximately 7 minutes long. SOAs were blocked and their order of presentation was counterbalanced. Within each task block, conditions were presented in an event-related fashion.

**Figure 1:**
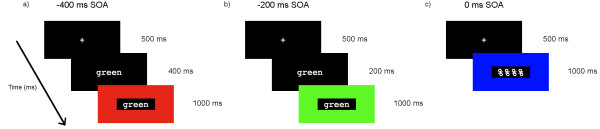
**Illustration of paradigm.** Examples of **a)** a −400 ms SOA incongruent condition; **b)** a −200 ms SOA congruent condition; and **c)** a 0 ms SOA control condition. Duration (ms) of each stimulus is indicated to the right.

Each SOA consisted of 120 trials (30 each of congruent, control, incongruent and null events). In the −400 ms SOA, the word appeared on the screen alone for 400 ms before being surrounded by the coloured rectangle (see Figure 1). In the −200 ms SOA, the word appeared for 200 ms before being surrounded by the colour. In the 0 ms SOA, both stimuli appeared simultaneously. Once both word and colour stimuli had appeared, both remained on the screen for 1000 ms. In null-event trials, a non-bold fixation cross remained on the screen for 750 ms. Each trial was followed by an ISI fixation screen with a non-bold fixation cross, varying from 1500–2900 ms in 200-ms intervals (average 2200 ms). The trial order was pseudo-randomly presented such that each trial type (congruent, control, incongruent) was followed equally often by a null event trial, and there were no occurrences of the same trial type occurring twice in a row throughout a block.

### fMRI scan procedure and pre-processing

Structural and functional MRI scans were acquired using a Philips Achieva 3.0 Tesla scanner at the Sir Peter Mansfield Magnetic Resonance Centre at the University of Nottingham. A sagittal T1-weighted volumetric sequence (TR 7600 ms, TE 2.3 ms, flip angle 8 degrees, NSA 1.0, FOV 256 mm, 256 × 256 matrix, 1.0 mm slice thickness, no gap, 184 slices) was acquired as a structural reference scan. fMRI was collected using gradient-echo EPI BOLD (echoplanar blood oxygenation level dependent) pulse sequences (TR 2500 ms, TE 40 ms, flip angle 90 degrees, 1 NSA, SENSE factor 2.3, resolution 3 × 3 × 3 mm, 38 slices of 3 mm thickness, no gap, FOV 240 mm, matrix size 80 × 80).

All pre-processing and data analyses were performed using SPM8 (Wellcome Trust Centre for Neuroimaging, “Statistical Parametrical Mapping, SPM8”, http://www.fil.ion.ucl.ac.uk/spm/). For each subject, functional images were spatially realigned to the first volume of the first run to account for motion during the scan. The anatomical scan was then co-registered to a mean EPI image of the realigned functional scans. The original anatomical scan was segmented using DARTEL [53] into grey matter (GM), white matter (WM) and cerebro-spinal fluid (CSF) in order to create a template of transformation parameters for normalizing the anatomical image to an MNI template brain. Functional and structural images were then normalized using these parameters. The normalized functional images were spatially smoothed using an 8 mm FWHM isotropic Gaussian kernel.

### fMRI analyses

Vectors of stimuli onsets were created for each trial type. For the analyses addressing the question of SOA effects on neural interference and facilitation, onsets were defined as the onset time of the colour stimulus, which was the second stimulus presented in the negative SOAs and therefore corresponded to the onset of conflict. For the analyses investigating global SOA effects (response priming effects in negative SOAs and effects of blocked SOAs on strategic attentional orientation), onsets were defined as the onset time of the first stimulus presented (i.e. in negative Stroop SOAs, onset of the word stimulus). Behavioural errors and outliers were included as additional conditions in the model specification. Six realignment parameters from the realignment step of pre-processing were also included as covariates. The stimuli onset vectors were convolved using a canonical HRF plus the temporal derivative. Statistical analyses based on general linear modeling (GLM) were then performed by multiple linear regression of the signal time course in each voxel. The three Stroop runs (0 ms, -200 ms, and −400 ms SOAs) were modeled together in the same design matrix. Three directional contrasts of interest were performed for each SOA (Stroop: incongruent > congruent; interference: incongruent > control; facilitation: control > congruent). Both the nonderivative and the temporal derivative were included when defining the contrasts, in order to avoid amplitude bias and to capture the temporal shift in the hemodynamic response function as a result of negative SOA presentation [54]. Percent signal change was calculated using Marsbar [55], and significant regions were labeled using the WFU PickAtlas package [56] and confirmed using the Talairach Client [57,58]. In all tables, regions and Brodmann areas (BA) for the entire cluster are listed, while Z-score and MNI coordinates are reported for the peak of the cluster. In identifying significant areas of activation in all analyses, an uncorrected *p*-value of *p* < 0.001 for the height (intensity) threshold of each activated voxel was used, with an extent threshold (cluster size) of 30 voxels.

## Results

### Behavioural data

**Figure 2:**
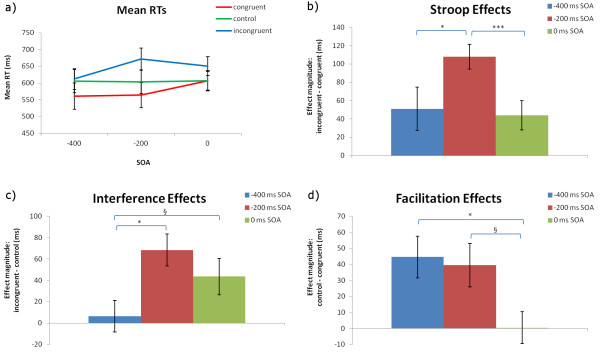
**Behavioural data. a)** Mean RTs for each congruency and SOA (standard error in parentheses). **b)** Stroop; **c)** interference; and **d)** facilitation effects, with significant differences between SOAs, as determined by paired-sample *t*-tests (two-tailed), indicated (§ = trend, *p* < 0.1; * = *p* < 0.05; ** = *p* < 0.01; *** = *p* < 0.001).

Incorrect responses (5.9%) and outliers (RTs of less than 250 or greater than 2000 ms; 0.3%) were removed before analyses. Because error rates were very low, no error analyses were performed. The mean RTs and magnitudes of Stroop (incongruent minus congruent RTs), interference (incongruent minus control (‘%%%%’)), and facilitation (control minus congruent) effects are shown in Figure 2. A 3 (congruency) × 3 (SOA) ANOVA showed a main effect of congruency (*F*(2,26) = 20.27, *p* < 0.0001) but not of SOA (*F*(2,26) = 1.75, *p* = 0.19), and an interaction of SOA and congruency (*F*(4,52) = 4.86, *p* < 0.01). Significant Stroop effects occurred in the −400 ms (*t*(13) = 2.17, *p* < 0.05), -200 ms (*t*(13) = 8.06, *p* < 0.0001) and 0 ms SOAs (*t*(13) = 2.76, *p* < 0.05). Significant interference occurred in the −200 ms (*t*(13) = 4.60, *p* < 0.001) and 0 ms SOAs (*t*(13) = 2.56, *p* < 0.05), and significant facilitation in the −400 ms (*t*(13) = 3.42, *p* < 0.01) and −200 ms SOAs (*t*(13) = 2.91, *p* < 0.05). The −200 ms SOA generated the largest Stroop (108 ms, *SE* = 13 ms; Figure 2b) and interference (68 ms, *SE* = 15; Figure 2c) effects. Similar facilitation effects occurred at the −400 ms SOA (45 ms, *SE* = 13 ms) and the −200 ms SOA (39 ms, *SE* = 14 ms), whereas facilitation was absent in the 0 ms SOA (0.4 ms, *SE* = 10 ms; Figure 2d).

### fMRI data

#### Trial-specific effects of SOA

As outlined in the Introduction, this study first investigated how SOA affected the neural representations of conflict and facilitation effects. Before comparing SOAs to address this question, the contrasts of interest (Stroop, interference, and facilitation) were investigated for each SOA individually using one-sample *t*-tests (Table 1).

**Table 1:** Main effects of interest (clusters > 30 voxels) for each SOA

**SOA**	**Contrast**	**Region**	**BA(s)**	**MNI coordinates**			**Cluster size**	**Peak Z-score**
				**x**	**y**	**z**		
0 ms SOA	Stroop effect	L precentral gyrus/postcentral gyrus	6/44	-56	-4	44	613	4.70
		Medial frontal gyrus/ACC/RCZ	8/6/32	4	26	46	209	3.88
		L inferior/superior parietal lobule/angular gyrus	40/7	-34	-50	46	185	4.07
		L middle temporal gyrus/angular gyrus/superior parietal lobule	39/7/19	-36	-72	26	171	4.08
		L middle/inferior frontal gyrus	46/10	-40	38	22	159	4.90
		L superior temporal gyrus/supramarginal gyrus	22/40	-58	-36	22	117	4.40
		R precuneus/superior parietal lobule	7	12	-70	44	90	3.85
		R superior temporal gyrus	41/13	48	-38	14	60	4.03
		L paracentral lobe/precentral gyrus/medial frontal gyrus	6	-6	-30	60	54	3.67
		R inferior parietal lobe/superior temporal gyrus/supramarginal gyrus	40	66	-40	22	49	4.24
		Posterior cingulate	29/23	-2	-40	18	48	3.81
		R inferior temporal gyrus/lateral occipitotemporal gyrus	20/37	50	-54	-14	47	3.62
		R inferior/superior parietal lobule/angular gyrus	40	32	-54	38	45	3.59
		L medial dorsal nucleus/thalamus	--	-10	-20	12	41	3.68
		R putamen/caudate nucleus	--	16	2	10	30	3.73
	Interference effect	L superior parietal lobule/angular gyrus	7/40	-12	-60	54	1082	4.75
		L middle frontal gyrus	8/9	-50	18	38	267	4.00
		L inferior/middle frontal gyrus	46	-50	30	16	198	4.12
		L medial frontal gyrus/ACC/RCZ	8/32	-4	28	40	173	4.13
		R paracentral lobe/precentral/postcentral gyrus	5/6	12	-38	52	100	3.76
		L cingulate/medial frontal gyrus	31/6	-16	-20	44	83	3.71
		R precuneus/superior parietal lobule	7	18	-74	46	81	3.67
		L middle frontal gyrus	6	-40	2	52	67	4.11
		L fusiform gyrus/lateral occipitotemporal gyrus	37/19	-44	-50	-14	66	3.79
		L superior/middle temporal gyrus	39	-56	-62	18	44	3.99
		R caudate nucleus	--	14	6	18	43	4.11
		R supramarginal gyrus/angular gyrus	40	58	-46	24	43	3.74
		L middle temporal gyrus/middle occipital gyrus	39	-52	-72	22	36	4.55
	Facilitation effect	No voxels surviving thresholding	--	--	--	--	--	--
-200 ms SOA	Stroop effect	R inferior frontal gyrus/insula	45/13/47	34	28	6	285	4.87
		L precentral gyrus/inferior frontal gyrus	6/44/9	-52	8	30	211	3.90
		L superior/medial frontal gyrus /ACC/RCZ	8/32/6	-8	10	48	80	4.27
		L inferior frontal gyrus/insula	45/13/47	-30	28	4	74	3.68
		R anterior/middle cingulate	32	8	38	22	63	3.84
		R middle temporal gyrus	37	54	-54	0	55	3.87
		R medial frontal gyrus/anterior cingulate	10	8	56	8	55	3.61
		R superior frontal gyrus/SMA	6	8	12	50	37	3.85
		L superior/inferior parietal lobe	7	-24	-56	42	36	3.67
		L inferior parietal lobe	40/2	-44	-38	46	35	3.89
	Interference effect	L inferior/middle frontal gyrus	9/46	-48	16	24	148	3.91
		R precuneus/superior parietal lobule	7	6	-66	42	102	3.87
		R precentral gyrus/inferior frontal gyrus	44/47	50	16	-8	70	4.04
		R superior parietal lobe/angular gyrus	7	34	-56	48	37	3.76
		R middle/inferior frontal gyrus	9/45	50	20	28	35	3.62
	Facilitation effect	No voxels surviving thresholding	--	--	--	--	--	--
-400 ms SOA	Stroop effect	L middle/superior frontal gyrus	6	-20	-8	62	61	4.27
	Interference effect	R thalamus	--	14	-22	10	236	4.67
		R inferior frontal gyrus	44/45/47	50	14	-6	133	4.48
		L thalamus	--	-10	-22	4	79	4.21
		R middle/superior temporal gyrus	22	58	-32	2	77	4.79
	Facilitation effect	No voxels surviving thresholding	--	--	--	--	--	--

Legend: Regions and Brodmann areas (BA) for the entire cluster are listed. Cluster size reported is number of voxels. Z-score and MNI coordinates are taken from the peak of the cluster.

**Figure 3:**
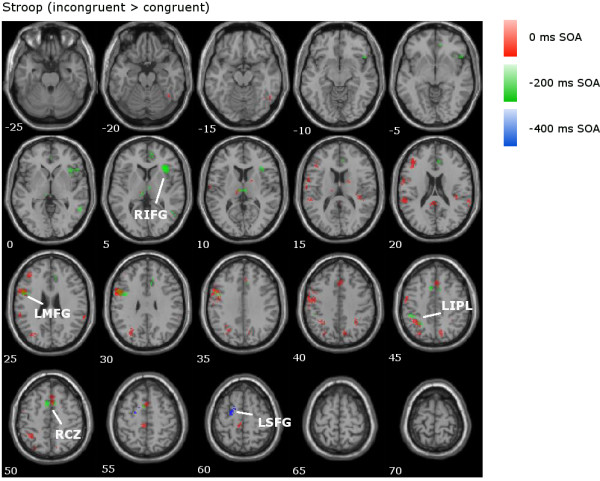
**Stroop contrast in the fMRI data.** Overlaid contrasts for the Stroop comparison (incongruent > congruent) for all SOAs, with clusters of interest labelled (LIPL = left inferior parietal lobe; LSFG = left superior frontal gyrus). Axial slices are shown from z = −25 to z = 70.

**Figure 4:**
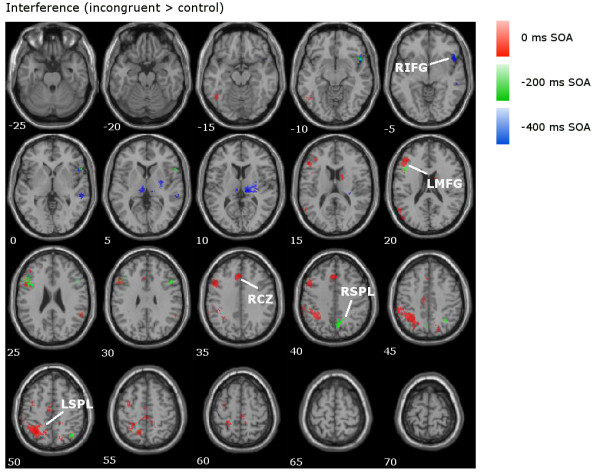
**Interference contrast in the fMRI data.** Overlaid contrasts for the interference comparison (incongruent > control) for all SOAs, with clusters of interest labelled (LSPL = left superior parietal lobe; RSPL = right superior parietal lobe).

**Figure 5:**
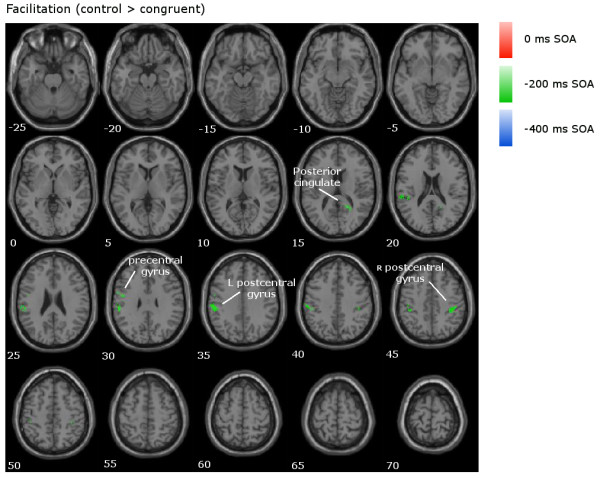
**Facilitation contrast in the fMRI data.** Overlaid contrasts for the interference comparison (control > congruent) for all SOAs, with clusters of interest labelled.

In the 0 ms SOA (shown in red in Figures 3, 4 and 5), the Stroop contrast revealed activation in cognitive control areas in the prefrontal and parietal cortices such as the LMFG (BAs 6/10), bilateral superior parietal lobes/angular gyri (BAs 7/40), LIFG (BA 46), posterior cingulate (BA 23) and ACC/RCZ (BAs 6/8/32), as well as subcortical activation in the left thalamus and right caudate nucleus (Table 1 and Figure 3). Similar areas were activated in the interference contrast (Figure 4): the bilateral superior parietal lobes/angular gyri (BA 40), LMFG (BAs 6/9), LIFG (BA 46), left ACC/RCZ (BA 8/32), and right caudate. The facilitation contrast showed no significant areas of activation for the 0 ms SOA (Figure 5).

In the −200 ms SOA (shown in green in Figures 3, 4 and 5), the Stroop contrast revealed activation in similar cognitive control areas as the 0 ms SOA: the bilateral insula/IFG (BAs 13/47), LIFG/LMFG (BAs 44/6/9), right MFG (BA 6), ACC/RCZ (BAs 6/32), left superior parietal lobe/angular gyrus (BAs 7/40), and the right middle temporal gyrus (BA 37; Table 1 and Figure 3). In the interference contrast (Figure 4), significant clusters of activation were observed in the bilateral IFG/MFG (BAs 9/44/45/46/47), and right superior parietal lobule (BA7). The facilitation contrast showed significant activation in the left and right postcentral gyrus (BAs 2/40), the left precentral gyrus (BAs 6/4), and the right posterior cingulate (BA 30; Figure 5).

In the −400 ms SOA (shown in blue in Figures 3, 4 and 5), the Stroop contrast revealed a cluster in the left middle/superior frontal gyrus (BA 6; Table 1, Figure 3). The interference contrast (Figure 4) revealed significant activation in the bilateral thalamus, right IFG (BA 47), and right superior temporal gyrus (BA 22). The facilitation contrast showed no significant activation (Figure 5).

#### SOA modulation of neural interference and facilitation effects

To investigate trial-specific effects of SOA on conflict and facilitation, three second-level ANOVAs were performed (for the Stroop, interference, and facilitation effects, respectively) by entering the first-level effect contrasts for each SOA into a 1-way ANOVA with three levels (SOA; Table 2).

**Table 2:** Local effects of SOA in the fMRI data

**Contrast**	**Region**	**BA**	**MNI coordinates**			**Cluster size**	**Peak Z-score**
			**x**	**y**	**z**		
Stroop effect	No voxels surviving thresholding	--	--	--	--	--	--
Interference effect	R precuneus/superior parietal lobe	7	12	-62	40	282	4.36
	L medial frontal gyrus/RCZ	8	-6	28	38	101	3.80
	R paracentral lobule	5/3	16	-40	54	65	4.30
	R superior frontal gyrus	9	22	48	36	54	4.19
Facilitation effect	R inferior parietal lobule/postcentral gyrus	40/2	38	-32	44	32	3.66

Legend: Results of the 3-way (SOA) ANOVAs identifying local effects of SOA on Stroop, interference, and facilitation effect magnitude, with a threshold of *p* < 0.001 and clusters > 30 voxels.

**Figure 6:**
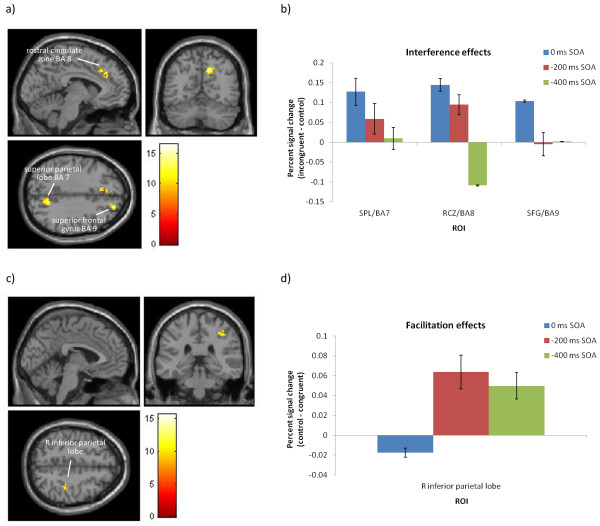
**Interaction of interference and SOA in the fMRI data.** Results of the 3-way ANOVA identifying significant interactions of **a)** interference and **c)** facilitation magnitude with SOA, with ROIs indicated. Panels to the right show the percent signal change effect in **b)** interference (incongruent signal change minus control signal change) and **d)** facilitation (control signal change minus congruent signal change) for each ROI and SOA.

The Stroop ANOVA revealed no significant clusters of activation. The interference effects elicited a main effect of SOA in three areas of the control network (Figure 6a): the RCZ (BA 8), right superior frontal gyrus (BA 9), LMFG (BA 6), and right superior parietal lobule (BA 7), as well as a cluster in the right paracentral lobule (BA 5/3). To further investigate how SOA modulated interference effects in these regions, the percent signal change for each condition was extracted from these three ROIs. The percent signal change interference effects (i.e. incongruent signal change minus control signal change; Figure 6b) demonstrated the largest neural interference effects in these areas in the 0 ms SOA. Finally, the facilitation effects elicited a main effect of SOA in the right inferior parietal lobe (BAs 40/2; Table 2 and Figure 6c). To investigate the direction of these effects the percent signal change was extracted for this cluster (Figure 6d). Percent signal change demonstrated that in the negative SOAs the control stimuli had greater percent signal change than congruent (−200 ms SOA: control = 0.20, congruent = 0.14; -400 ms SOA: control = 0.14, congruent = 0.09), whereas in the 0 ms SOA the congruent and control stimuli elicited similar levels of percent signal change (0 ms SOA control = 0.01, congruent = 0.02).

#### Global (block-wide) effects of SOA

As discussed in the Introduction, SOA was predicted to create global (i.e. block-wide or conflict-independent) effects of response priming in negative SOAs [40] and of attentional control due to blocked SOA presentation [48]. These effects of SOA were expected when evaluating block-wide SOA effects (collapsing over congruencies), as well as when directly comparing congruencies between SOAs. For example, block-wide attentional orientation should be present in all congruencies, leading to differences even when comparing control conditions between SOAs.

**Figure 7:**
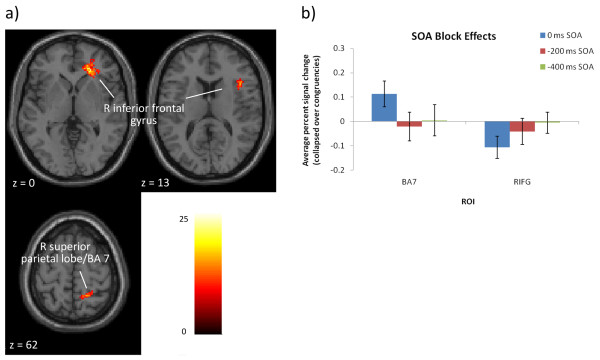
**Global SOA effects in the fMRI data.** Results of the 3-way ANOVA collapsing across congruency to investigate global (i.e. block-wide) SOA effects. **a)** Axial slices presented at three z-coordinates to illustrate two distinct clusters in the right inferior frontal gyrus, as well as a cluster in the superior parietal lobe. **b)** The percent signal change for each ROI and SOA, collapsed over congruency (the RIFG percent signal change was extracted from the larger cluster at z = 0).

To investigate block-wide SOA effects, each SOA was first collapsed over congruencies (contrasted with null-event trials: (incongruent, control, congruent) > null) and entered into a 1-way ANOVA with three levels (SOA). Two main regions emerged that were sensitive to global SOA effects: the RIFG (BAs 45/47; part of a RIFG cluster also extended into the ACC/BA 32) and the right superior parietal lobe (BA 7; see Figure 7 and Table 3). The percent signal change for each SOA was also extracted from these two regions, which revealed larger overall effects for the 0 ms SOA (when collapsed across congruencies: Figure 7b). A block effect of SOA was confirmed by the presence of differences in the overall level of activation in each SOA.

**Table 3:** Global effects of SOA in the fMRI data

**Contrast**	**Region**	**BA(s)**	**MNI coordinates**			**Cluster size**	**Peak Z-score**
			**x**	**y**	**z**		
Block-wide SOA effect	R ACC/inferior frontal gyrus	32/47	16	38	0	310	5.32
	R inferior frontal gyrus	45	42	20	10	104	4.38
	R superior parietal lobe	7	18	−48	62	75	4.41

Legend: Results of the 3-way ANOVA identifying global effects of SOA manipulation by collapsing across congruency in each SOA block, with a threshold of *p* < 0.001 and clusters > 30 voxels.

To further investigate global effects of SOA, each congruency was compared between SOAs using two-sample *t*-tests (e.g. -400 ms congruent > 0 ms congruent; Table 4). Due to the pre-exposure of the word in negative SOAs, visual activation was expected in these conditions compared to the 0 ms SOA. The data confirmed this prediction: the −400 ms SOA showed more activation than the other SOAs across all congruencies in medial, lateral, and inferior areas of the occipitotemporal gyrus (BAs 17/18/19) and the −200 ms SOA incongruent condition activated the lateral occipitotemporal gyrus (BA 36) compared to the 0 ms SOA. All subsequent analyses focused on activation outside of the occipital cortex. Of particular interest were SOA effects in the congruent and control conditions, which would suggest a conflict-independent effect of SOA on response priming and/or strategic attention.

**Table 4:** Between-condition comparisons across SOAs of the Stroop task (clusters > 30 voxels) for each congruency condition

**Congruency condition**	**Contrast**	**Region**	**BA(s)**	**MNI coordinates**			**Cluster size**	**Peak Z-score**
				**x**	**y**	**z**		
Congruent	0 ms > -200 ms	No voxels surviving thresholding	--	--	--	--	--	--
	-200 ms > 0 ms	L insula	13	-40	4	-8	85	3.76
		L insula/postcentral gyrus	13/45	-42	-12	20	45	3.98
		L superior/middle frontal gyrus	9	-14	50	22	44	3.76
		L inferior frontal gyrus	45/47/13	-48	16	4	43	3.91
	0 ms > -400 ms	R superior parietal lobule/postcentral gyrus	7	22	-46	62	46	3.78
		L postcentral gyrus	2/40	-40	-36	60	39	3.77
	-400 ms > 0 ms	L insula/inferior frontal gyrus/precentral gyrus	13/38	-46	12	2	105	3.91
		L middle/inferior occipital gyrus	18	-26	-84	-2	75	3.85
	-200 ms > -400 ms	No voxels surviving thresholding	--	--	--	--	--	--
	-400 ms > -200 ms	No voxels surviving thresholding	--	--	--	--	--	--
Control	0 ms > -200 ms	No voxels surviving thresholding	--	--	--	--	--	--
	-200 ms > 0 ms	L parahippocampal gyrus	--	-32	-24	-14	77	4.22
		L superior frontal gyrus	9	-14	46	22	45	4.77
		L ACC	32	-14	22	18	34	4.21
		L insula/postcentral gyrus	13	-40	-10	22	34	4.04
	0 ms > -400 ms	No voxels surviving thresholding	--	--	--	--	--	--
	-400 ms > 0 ms	L lingual gyrus/medial occipitotemporal gyrus	18/17	-26	-84	-2	452	4.71
		R lingual gyrus/lateral occipitotemporal gyrus	18	22	-76	-2	92	4.14
		R inferior frontal gyrus/insula	47/45	44	16	-8	87	3.91
		L inferior frontal gyrus/insula/inferior temporal gyrus	47/38	-40	14	-12	51	3.89
		R fusiform gyrus/lateral occipitotemporal gyrus	19	38	-68	-8	46	3.86
		L middle/inferior frontal gyrus	10/47	-32	36	8	43	4.19
		R parahippocampal gyrus/medial occipitotemporal gyrus	19	26	-54	-2	33	3.77
	-200 ms > -400 ms	R posterior cingulate	23/29	4	-36	18	89	4.12
		R posterior cingulate gyrus/angular gyrus	31	24	-44	38	82	4.88
	-400 ms > -200 ms	No voxels surviving thresholding	--	--	--	--	--	--
Incongruent	0 ms > -200 ms	No voxels surviving thresholding	--	--	--	--	--	--
	-200 ms > 0 ms	L posterior cingulate gyrus	31	-18	-40	34	73	4.54
		L parahippocampal gyrus/lateral occipitotemporal gyrus	36	-30	-32	-10	69	4.36
	0 ms > -400 ms	R posterior cingulate gyrus	31	24	-44	38	40	4.27
		L cuneus/superior occipital gyrus	19/18	-4	-88	30	38	4.44
	-400 ms > 0 ms	L middle occipital gyrus	18	-26	-84	-2	35	3.74
	-200 ms > -400 ms	R posterior cingulate gyrus	31	24	-44	38	72	4.47
	-400 ms > -200 ms	R parahippocampal gyrus	28	20	-14	-22	36	4.42

The full results are presented in Table 4. To summarize the most important effects, which will be further interpreted with specific regards to response priming effects and strategic attentional control in the Discussion, the −200 ms SOA elicited stronger activation compared to the 0 ms SOA for the congruent and control conditions in the left superior/middle frontal gyrus (BA 9). The −400 ms SOA control condition activated the RIFG to a greater extent than the 0 ms SOA control condition. The −200 ms SOA incongruent and control conditions activated the posterior cingulate more than these congruencies in other SOAs. The congruent and incongruent conditions showed enhanced right superior parietal lobe (BA 7) and posterior cingulate activation in the 0 ms SOA compared to the −400 ms SOA. Finally, the −400 ms SOA congruent and control conditions, and the −200 ms SOA congruent condition, activated the LIFG to a greater extent than the corresponding congruencies in the 0 ms SOA.

## Discussion

The current study employed fMRI to investigate for the first time how the executive control network is modulated by SOA in a Stroop task. Of particular interest were 1) the neural effects of SOA on interference and facilitation effects; 2) response priming in negative SOAs; and 3) the effects of blocked SOA presentation on strategic orientation of attention. To briefly summarize the results that will be discussed at length in the next sections, four areas in the executive control network were sensitive to trial-specific SOA effects on interference. An overall ANOVA investigating the global, congruency-independent effects of SOA demonstrated that the RIFG was sensitive to response priming effects in negative SOAs, whereas the right superior parietal lobe (BA 7) was sensitive to attentional effects of blocked SOA presentation.

### SOA modulation of interference and facilitation effects

Previous work with SOA manipulation in the Stroop task has documented varying amounts of interference and facilitation in each SOA. Behaviourally, the current data replicated prior observations that, in a manual task, peak interference occurred at the −200 ms SOA and was also significant at the 0 ms SOA [38-40]. Facilitation was similar between the −400 ms and −200 ms SOAs, which also replicates previous literature [38,41,43,44]. Importantly, the effects of SOA on the magnitudes of interference and facilitation effects suggest differences in executive control strategies or recruitment in each SOA, which was investigated with fMRI for the first time in the current study.

Analyses indicated that areas of the brain participating in Stroop effects were not strongly modulated by SOA. However, three areas of the cognitive control network were sensitive to the effects of SOA on interference: the right superior parietal lobe (BA 7), RCZ (BA 8), and superior frontal gyrus (BA 9). Percent signal change analyses indicated that these areas showed greater BOLD change for the 0 ms and −200 ms SOAs than the −400 ms SOA, suggesting sensitivity to the magnitude of cognitive conflict. Specifically, this indicates that areas involved in performance monitoring (RCZ), conflict resolution (superior frontal gyrus/BA 9), and task-relevant attentional control (superior parietal lobule/BA 7) were most affected by interference during simultaneous stimuli presentation in the 0 ms SOA. As this SOA showed comparatively smaller behavioural interference effects, the stronger recruitment of these areas may reflect more efficient conflict processing. In contrast, the reduced activation in the −200 ms SOA illustrates that conflict resolution mechanisms were not engaged as efficiently, generating larger behavioural effects. In sum, this demonstrates that SOA significantly affected the recruitment of the cognitive control network during interference, as predicted.

When investigating Stroop, interference, and facilitation effects in each SOA individually, the 0 ms SOA showed a traditional recruitment of the executive control network for Stroop and interference effects, including the RCZ, LMFG, LIFG, and right superior parietal lobe, in line with previous literature [1,3,4,12,21,23,24]. The −200 ms SOA activated these same areas but to a lesser extent, again suggesting a less-efficient recruitment of cognitive control which generated increased behavioural interference. Therefore the two most cognitively-demanding SOAs activated a similar neural network, but the amount of activation was modulated by SOA.

Despite the relatively reduced activation in the conflict contrasts of the −200 ms SOA, when directly comparing the congruency conditions this SOA showed heightened ACC and LMFG (BA 9) activation in all congruencies, including the control condition. Previous research has reported that the ACC and prefrontal cortex are sensitive to the amount of conflict in a task [29] and that activation can be enhanced with task difficulty across the entire task rather than on a trial-by-trial basis [59]. The observed ACC and LMFG activation therefore suggests that cognitive control was enhanced throughout the −200 ms SOA block and in all congruencies due to the heightened cognitive demands in this SOA.

Overall, there was a disparity between the behavioural and neural effects: the 0 ms SOA elicited stronger brain activity yet experienced smaller behavioural conflict effects, while the opposite was true for the −200 ms SOA. This negative association of behavioural and neural responses has been reported previously [60-63] and suggests that successful cognitive control requires more extensive activation of the executive control network to reduce behavioural conflict effects.

The block-wide facilitation ANOVA also demonstrated that a cluster in the right inferior parietal lobe (BA 40) was sensitive to SOA effects on facilitation, and percent signal change analyses confirmed that this area showed greater signal change for the −200 and −400 ms SOAs compared to the 0 ms SOA. This mirrors the behavioural data, which showed large facilitation effects for the negative SOAs but virtually no facilitation for the 0 ms SOA, and also supports previous literature finding similar facilitation effects across negative SOAs [38,41,43,44].

When extracting the percent signal change from this cluster in the facilitation effects, the results showed that the negative SOAs showed larger signal change in right BA 40/2 for the control condition than the congruent condition, whereas the 0 ms SOA showed similar levels of signal change for both congruencies. As mentioned in the Introduction, the parietal lobes are involved in top-down attentional control towards the task-relevant target or attribute [17-20]. It may be that in negative SOAs, pre-exposure of the control stimulus allows the semantic system to evaluate the stimulus and determine that the symbol string has no meaning, such that when the colour appears attention can be more efficiently directed to the target stimulus; this could explain the greater activation of the parietal lobe in response to the control stimulus compared to the congruent stimulus. In contrast, in the 0 ms SOA, simultaneous presentation of stimuli requires that the word be evaluated at the same time the colour is being processed, which may interrupt this efficiency of the parietal lobe. This is a tentative interpretation, however, and more research is needed to fully evaluate the neural correlates of SOA effects on facilitation. Nevertheless, the current results confirm that SOA manipulation does modulate facilitation effects, both behaviourally and in the brain.

In sum, the cognitive control network was sensitive to trial-specific effects of SOA on interference. Specifically, three regions of the network were most active in the 0 ms SOA, leading to correspondingly smaller behavioural interference effects. In contrast, the −200 ms SOA experienced comparatively less neural activation, suggesting less-efficient cognitive control which led to larger behavioural interference effects. This therefore demonstrates that SOA modulates the conflict-processing demands of the executive control network and suggests that short pre-exposure of the word in the −200 ms SOA disrupts the efficient processing of this system.

### Response priming effects in negative SOAs

Appelbaum et al. [40] have suggested that negative SOAs create a response priming effect by pre-activating response selection, which generates larger behavioural interference and facilitation effects compared to the 0 ms SOA. This study explored the neural representation of these response priming effects in the −200 ms and −400 ms SOAs. The block-wide SOA analysis identified two regions that were modulated by the global effects of SOA: the RIFG and the right superior parietal lobe. As will be argued here, the RIFG was involved in response priming effects.

In the SOA-specific analyses, the Stroop and interference contrasts in the −200 ms SOA elicited RIFG activation to a greater extent than the 0 ms SOA; additionally, the −400 ms SOA activated the RIFG in the interference contrast. As mentioned in the Introduction, the RIFG has been implicated in response inhibition (i.e. inhibiting pre-potent motor responses, as in a no-go paradigm; [13-16]). The activation of this area in negative SOAs suggests its involvement in response priming effects; specifically, the fact that RIFG activation occurred in Stroop and interference contrasts in negative SOAs suggests that this area is involved in applying response inhibition after incorrectly-primed response selection.

To illustrate, in incongruent conditions the pre-exposed word primes (incorrect) response selection, which must then be overcome (via response inhibition mechanisms in the RIFG) to make a correct response to the colour. This would explain why the −200 ms SOA generates larger interference and facilitation effects: the need for response inhibition in incongruent conditions leads to longer incongruent RTs and consequently larger behavioural interference effects relative to the other conditions. In congruent conditions, however, the primed response preparation leads to faster RTs and increased behavioural facilitation effects. Response priming in the RIFG can therefore explain the larger interference and facilitation effects observed in the −200 ms SOA, as observed in the current data and in previous research [38-40].

In contrast, the −400 ms SOA generated large behavioural facilitation effects but no interference, which contradicts the proposal that response priming increases both interference and facilitation effects. In direct comparisons of the individual congruencies in the neural data, the −400 ms SOA showed more RIFG activation in the control condition compared to other SOAs. This suggests that the RIFG cannot purely reflect response inhibition in this SOA, because a response cannot be primed in the control condition as it does not contain semantic information.

Although the RIFG has been specifically implicated in response inhibition, previous investigations of the right posterior ventrolateral prefrontal cortex, which includes the RIFG, have indicated that this area is involved more generally in updating action plans, a function which includes, but is not limited to, response inhibition [13-16,49,64,65]. The current data in the −400 ms SOA support this more general role of the RIFG in action updating. To illustrate, although the pre-exposure of the word primes response selection, the long pre-exposure may allow sufficient time to fully inhibit the motor response, as the word is a non-target stimulus: this would explain the lack of behavioural interference in the −400 ms SOA. If the primed response is fully inhibited, this would also predict a reduction in facilitation effects; however, facilitation is *increased* in this SOA. Therefore in addition to response inhibition, the RIFG may also perform more general action updating, as proposed by previous literature, which readies the motor system to make a response. If response preparation mechanisms are primed in a −400 ms SOA, upon subsequent colour presentation the system benefits from the convergent information in the congruent condition (therefore generating large facilitation effects) but the incongruent condition does not cause any additional conflict (resulting in little or no interference). In both congruencies, similar brain regions are active, which may explain the lack of neural differences between these conditions in the current −400 ms SOA data.

Thus, the current data can be explained by assuming that in the −200 ms SOA the RIFG is engaged primarily for response inhibition in incongruent conditions, as a result of the response priming effect, whereas in the −400 ms SOA the RIFG is involved in more general action updating. Importantly, in the −200 ms SOA the increased interference occurs because the response priming effect does not have enough time to be resolved. Lexical access occurs approximately 200 ms after word onset (e.g. [66,67]), meaning that the colour appears at the same approximate time that semantic activation occurs in this SOA, leaving little extra time for stimulus suppression before conflict arrives. As a result, there is not enough time to overcome the response priming in the incongruent condition before the colour arrives, creating conflict and requiring the RIFG to perform response inhibition. In contrast, in the −400 ms SOA there is ample time for both semantic activation of the word and subsequent suppression of the primed response (via the RIFG), which explains the lack of behavioural interference. As well as inhibiting the primed response, the RIFG also performs a more general function of action updating, priming the system to make a motor response. This is a tentative explanation, as the RIFG has also been implicated in other cognitive functions such as reorienting [68], the detection of salient cues [15], and stopping motor actions [27]. However, the current data fits best with an explanation of the RIFG as involved in response inhibition and updating action plans [13-16,49,64,65]. Therefore the current data supported Appelbaum et al. [40]’s proposal of response priming with word pre-exposure and also provided additional knowledge of how this mechanism functions in each SOA.

### Effects of blocked SOA presentation

The current study additionally investigated whether blocked SOA presentation would create a global effect of attentional orientation such that the temporal predictability could be used to direct attention to the upcoming target stimulus [41,48]. Such effects should be apparent across the entire block. As mentioned, the global (congruency-independent) analysis of SOA effects revealed two clusters of activation: the RIFG (which has been attributed to response priming effects of response inhibition and action updating [13-16,49,64,65]) and the right superior parietal lobe (BA 7). As BA 7 is involved in top-down attentional control [17-20] this area may have been sensitive to attentional control effects resulting from the temporal predictability of blocked SOAs.

It was expected that attentional control effects would be most prominent in negative SOAs, as the word pre-exposure might act as a temporal cue that the target colour would soon appear. However, the percent signal change analyses illustrated that BA 7 was most active for the 0 ms SOA. This could suggest that when stimuli are simultaneously presented, attention to the relevant stimulus (the colour) is enhanced in order to facilitate response selection. For example, Egner and Hirsch [61] have suggested that conflict resolution proceeds via amplification of task-relevant attributes; enhanced attentional control in the 0 ms SOA may therefore indicate a strategy of directed attention towards the colour in order to overcome the effects of the distracting word stimulus. In contrast, the pre-exposure of the word in the negative SOAs may disrupt this process, leading to less activation in BA 7. Interestingly, the enhanced activation of this area for the 0 ms SOA mirrors the findings of Appelbaum et al. [48], who reported a larger N_inc_ in the 0 ms SOA with blocked SOA presentation.

Block-wide strategic attention effects were also identified in the direct comparisons of congruencies across SOA blocks: specifically, the −200 ms SOA showed more activation in the posterior cingulate. While being assigned to a number of cognitive roles, one function of the posterior cingulate is in anticipating the need to spatially allocate attention [69]. This could suggest an attentional priming effect in the −200 ms SOA such that the short pre-exposure of the word acted as a cue for attentional engagement. It is unclear why a similar effect did not occur in the −400 ms SOA; one possibility is that the longer word pre-exposure allowed ample time for the suppression of the word information, so attentional allocation was not prioritized.

In summary, the right superior parietal lobe was sensitive to the effects of blocked SOA presentation, demonstrating that attentional control was modulated by the global effects of SOA. These effects were enhanced for the 0 ms SOA, which could suggest that attentional mechanisms of conflict resolution were engaged during simultaneous stimulus presentation in the 0 ms SOA. In addition, SOA effects in the posterior cingulate in the −200 ms SOA could reflect an anticipation of attentional control.

In general, the fact that global effects of SOA were observed in regions involved in attentional control supports the proposal of strategic orientation of attention with blocked SOA presentation. However, these are ultimately tentative interpretations in light of the fact that a mixed-SOA comparison condition was not included in order to fully test the effects of strategic attentional orientation. For example, if blocked SOA affects attentional orienting towards the relevant dimension, this effect should be diminished with mixed SOAs, leading to smaller interference at negative SOAs as compared to blocked presentation. Therefore mixed SOA presentation might lead to very different effects, both in the behavioural data and in the neural recruitment of the attentional control network [48]. As this was the first study to use the Stroop SOA paradigm with fMRI, the comparison of blocked vs. mixed SOAs, and how this paradigm choice affects the recruitment of conflict processing mechanisms, requires further exploration.

### Distractor suppression effects in negative SOAs

In addition to the reported effects of SOA on conflict processing, response priming, and attentional control, one additional finding was that the LIFG was generally more active across all congruencies for negative SOAs. Specifically, more LIFG activation was observed for the −400 ms SOA congruent and control conditions and the −200 ms SOA congruent condition as compared to the corresponding congruencies in the 0 ms SOA. Previous research has suggested that within the cognitive control network the LIFG performs suppression of irrelevant information (e.g. [12]); this finding of enhanced LIFG activation throughout the negative SOAs may therefore suggest a strategy of distractor suppression. For instance, at the time of word presentation in negative SOAs the word’s eventual congruency is unknown, as the colour has not yet appeared to cause conflict. Therefore the LIFG may be suppressing all pre-exposed information, as it is irrelevant to the task, in order to avoid potential conflict when the colour appears. Importantly, the control condition also elicited enhanced LIFG activation in negative SOAs, suggesting that this mechanism is neither conflict- nor linguistically-specific, but is a global strategy of task-irrelevant distractor suppression.

This proposal of a distractor suppression mechanism in negative SOAs suggests a strategy of proactive cognitive control, which draws a parallel to the *dual mechanisms of control* theory put forth by Braver and colleagues [70-72]. This theory proposes that cognitive control consists of two mechanisms: one reactive, which is a ‘late correction’ response that uses context information transiently to resolve conflict once it has occurred; and one proactive, which uses an ‘early selection’ strategy to actively sustain goal-relevant information and pre-emptively reduce control demands when conflict occurs. The fact that LIFG activation occurred across all congruencies in negative SOAs suggests a sustained activation of this structure, potentially through a mechanism of proactive cognitive control. In contrast, reactive control may be more characteristic of the 0 ms SOA, in which suppression must be activated anew on every trial. Although a tentative explanation, this proposal of distractor suppression by the LIFG suggests a proactive strategy employed to lessen the influence of the non-target stimulus and highlights the dynamic nature of the executive control system in response to various cognitive demands.

## Conclusions

In summary, the current data demonstrated both trial-specific and block-wide effects of SOA on the recruitment and behaviour of the executive control network. The network was activated to different extents in each SOA, with the largest neural interference effects in the 0 ms SOA compared to the −200 ms SOA. As the 0 ms SOA demonstrated relatively reduced behavioural interference effects, this more extensive neural activation suggests more efficient conflict processing, whereas in the −200 ms SOA this efficient processing was disrupted by the pre-exposure of the word. Response priming effects were localized to the RIFG; in the −200 ms SOA in particular, these effects can be explained by response inhibition in incongruent conditions in order to overcome the conflict created by the incorrectly primed response. In the −400 ms SOA, with longer word pre-exposure, the RIFG activation suggested more general response preparation and action updating, leading to increased behavioural facilitation but no interference. Strategic attention effects were localized to the right superior parietal lobe but were enhanced in the 0 ms SOA, suggesting that negative SOAs do not create a temporal cue; instead, attentional control mechanisms are enhanced in the 0 ms SOA to more efficiently deal with the conflict generated by simultaneous stimulus pre-exposure. Finally, word pre-exposure in the negative SOAs also appeared to recruit a proactive control strategy of distractor suppression, localized to the LIFG. As this was the first study to explore SOA modulation in the Stroop task with fMRI, there are ample possibilities for future research. However, the use of SOA manipulation has provided valuable information on the malleable and dynamic nature of cognitive control.

## Endnote

^a^This was a standard flanker task and did not employ SOA manipulation. As the focus of this data is on the modulation of the cognitive control network with SOA manipulation in the Stroop task, the data from the flanker task are not reported here.

## Competing interests

The authors declare that they have no competing interests.

## Authors’ contributions

EC participated in the conception and design of the study, collected the data, performed the statistical analysis, and drafted the manuscript. WVH participated in the conception and design, helped with interpretation of the data, and helped with revising the manuscript. Both authors read and approved the final manuscript.
